# The Role of α3β1 Integrin Modulation on Fabry Disease Podocyte Injury and Kidney Impairment

**DOI:** 10.3390/toxins15120700

**Published:** 2023-12-14

**Authors:** Bruna Bosquetti, Aline Aparecida Santana, Paulo Cézar Gregório, Regiane Stafim da Cunha, Guilherme Miniskiskosky, Julia Budag, Célia Regina Cavichiolo Franco, Edneia Amancio de Souza Ramos, Fellype Carvalho Barreto, Andréa Emilia Marques Stinghen

**Affiliations:** 1 Experimental Nephrology Laboratory, Basic Pathology Department, Universidade Federal do Paraná, Curitiba 81531-980, Brazil; brubosquetti@gmail.com (B.B.); aline07santana@gmail.com (A.A.S.); paulocezargregorio@gmail.com (P.C.G.); regidacunha@ufpr.br (R.S.d.C.); gui.miniski@gmail.com (G.M.); jbudag98@gmail.com (J.B.); crcfranc@ufpr.br (C.R.C.F.); edneiaama@ufpr.br (E.A.d.S.R.); 2 Internal Medicine Department, Division of Nephrology, Universidade Federal do Paraná, Curitiba 80060-900, Brazil; fellype.barreto@ufpr.br

**Keywords:** chloroquine, Fabry disease, podocyte injury

## Abstract

Podocyte dysfunction plays a crucial role in renal injury and is identified as a key contributor to proteinuria in Fabry disease (FD), primarily impacting glomerular filtration function (GFF). The α3β1 integrins are important for podocyte adhesion to the glomerular basement membrane, and disturbances in these integrins can lead to podocyte injury. Therefore, this study aimed to assess the effects of chloroquine (CQ) on podocytes, as this drug can be used to obtain an in vitro condition analogous to the FD. Murine podocytes were employed in our experiments. The results revealed a dose-dependent reduction in cell viability. CQ at a sub-lethal concentration (1.0 µg/mL) induced lysosomal accumulation significantly (*p* < 0.0001). Morphological changes were evident through scanning electron microscopy and immunofluorescence, highlighting alterations in F-actin and nucleus morphology. No significant changes were observed in the gene expression of α3β1 integrins via RT-qPCR. Protein expression of α3 integrin was evaluated with Western Blotting and immunofluorescence, demonstrating its lower detection in podocytes exposed to CQ. Our findings propose a novel in vitro model for exploring secondary Fabry nephropathy, indicating a modulation of α3β1 integrin and morphological alterations in podocytes under the influence of CQ.

## 1. Introduction

Fabry disease (FD) is a rare, hereditary, X-linked pathology caused by various mutations in the *GLA* gene, responsible for encoding the α-galactosidase A (α-GLA) enzyme [1,2]. The lack of this enzyme or its low activity results in the lysosomal accumulation of glycosphingolipids, notably globotriaocilceramide (Gb3) [3,4]. Progressive Gb3 deposition in lysosomes affects various cell types, leading to renal, cardiac, and/or cerebrovascular complications [5,6,7,8]. Renal involvement stands out as a major contributor to mortality and disability in FD. Classic manifestations include glomerular injury, glomerulosclerosis, proteinuria, and microalbuminuria, ultimately progressing to end-stage renal disease [6]. Podocytes, essential for the glomerular filtration process and having a structural role, are significantly affected by FD. Podocitary dysfunction or injury is a common consequence of various glomerulopathies and is linked to the gradual, asymptomatic decline in renal function [9,10].

FD closely mirrors the effects induced by chloroquine (CQ) or hydroxychloroquine (HCQ). These effects encompass signs, symptoms, and cellular changes observed in patients on chronic use of CQ or HCQ, including cardiomyopathy, proteinuria, and lysosomal inclusions in renal and cardiac tissues [11,12,13,14,15]. The amphiphilic nature of these molecules is responsible for these side effects, leading to their accumulation in lysosomes. This accumulation elevates lysosomal pH, alters the organelle’s membrane permeability, and causes enzymatic dysfunction [11,16,17]. Consequently, CQ and HCQ could potentially induce a phenocopy of FD, although their specific impact on podocytes remains unclear.

The pathophysiological mechanisms leading to podocyte dysfunction in the Glomerular Basal Membrane (GBM) remain unclear. It is established that the primary mediator of the binding between the actin cytoskeleton and GBM components is the α3β1 integrin [18]. In the present study, we aimed to assess the cellular and molecular mechanisms and effects of CQ on podocyte injury in vitro. Podocyte alterations have the potential to reduce adhesion to the GBM, a characteristic of podocyturia and proteinuria conditions in FD patients. Progress in this field may contribute to the development of new therapeutic interventions to enhance patient survival.

## 2. Results

### 2.1. CQ Decreases Podocyte Viability

**Figure 1:**
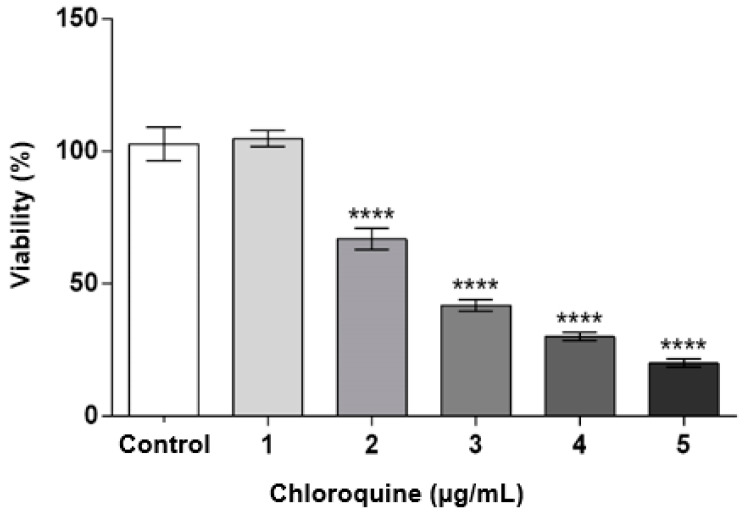
Dose–response curve of podocyte cells at different concentrations of CQ. Data were expressed as mean ± SEM of four independent experiments. The results were analyzed using ANOVA (*p* > 0.0001) and Dunnet’s multiple comparison test, in which **** *p* < 0.0001: 2 μg/mL vs. control + 3 μg/mL vs. control + 4 μg/mL vs. control + 5 μg/mL vs. control.

To determine the appropriate CQ concentration, a dose–response curve experiment was performed to evaluate cell viability in endothelial cells across different CQ concentrations (1.0, 2.0, 3.0, 4.0, and 5.0 μg/mL). CQ significantly decreased cell viability (*p* < 0.0001) at concentrations of 2.0, 3.0, 4.0, and 5.0 μg/mL (Figure 1). Notably, a concentration of 1.0 μg/mL CQ did not impact cell viability, and this concentration was chosen for subsequent experiments.

### 2.2. CQ Promotes Acid Organelle Accumulation

**Figure 2:**
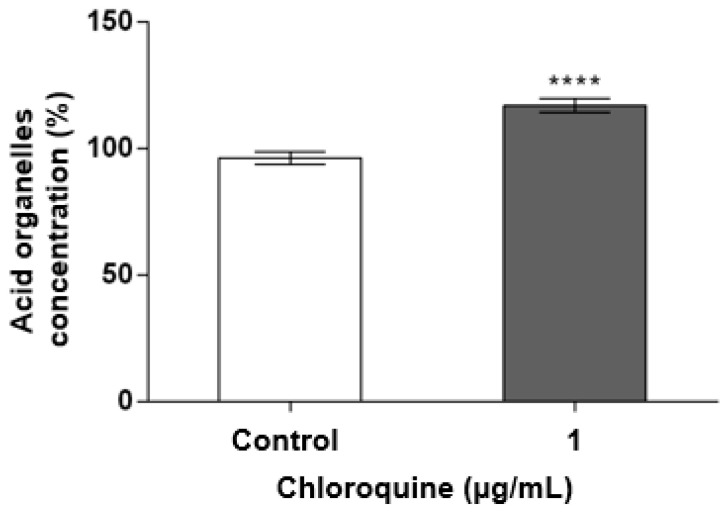
CQ induces increase in acid organelles. Podocytes were incubated with 1.0 μg/mL of CQ or vehicle control (culture medium) for 72 h. Acid organelles were assessed using NR and VC methods. Result is expressed as % of control (vehicle) and represents the mean ± SEM of three independent experiments. The results were analyzed using *t* test. **** *p* < 0.0001.

**Figure 3:**
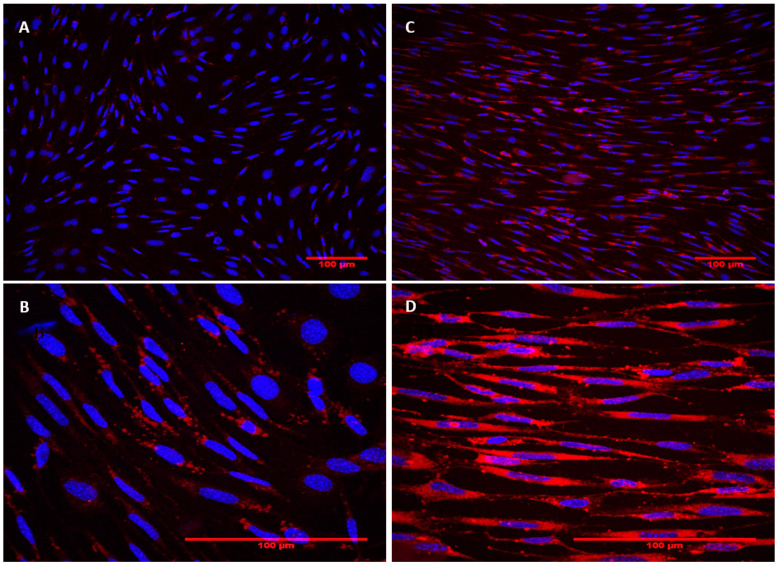
CQ induces increase in lysosomal inclusions. Podocytes were treated with CQ (1 µg/mL) or vehicle control (culture medium) for 72 h at 37 °C. The lysosomes (red) and nucleus (blue) were marked with the probes Lysotracker^®^ DND-99 and DAPI, respectively. (**A**) Control cells (treated with culture medium)—200× magnification; (**B**) control cells (treated with culture medium)—600× magnification; (**C**) treated cells—200× magnification; and (**D**) treated cells—600× magnification.

Once a non-lethal concentration of CQ was determined for the treatment of podocytes, the experiments were performed to demonstrate the increase in acid organelle accumulation (Figure 2) and the increase in lysosomal inclusions (Figure 3). A significant increase (*p* < 0.0001) in the amount and/or size of acidic organelles was observed at a concentration of 1.0 μg/mL (Figure 2). Figure 3 shows the markings for the lysosomes in red and the markings for the nucleus in blue. In Figure 3A,B, the lysosomal marking reflects the normal cellular constitution, with the lysosomes dispersed evenly in the cell cytoplasm. In Figure 3C,D, cell labeling becomes much more pronounced, demonstrating that there is a large increase in the number of lysosomes. These results confirm the lysosomal accumulation induced by CQ. Additionally, cells treated with CQ exhibited a more elongated appearance, fewer cell-to-cell interactions, and morphological changes in the nucleus. To further assess this, an ultrastructural analysis of the cells was performed.

### 2.3. CQ Promotes Podocyte Structural Changes

**Figure 4:**
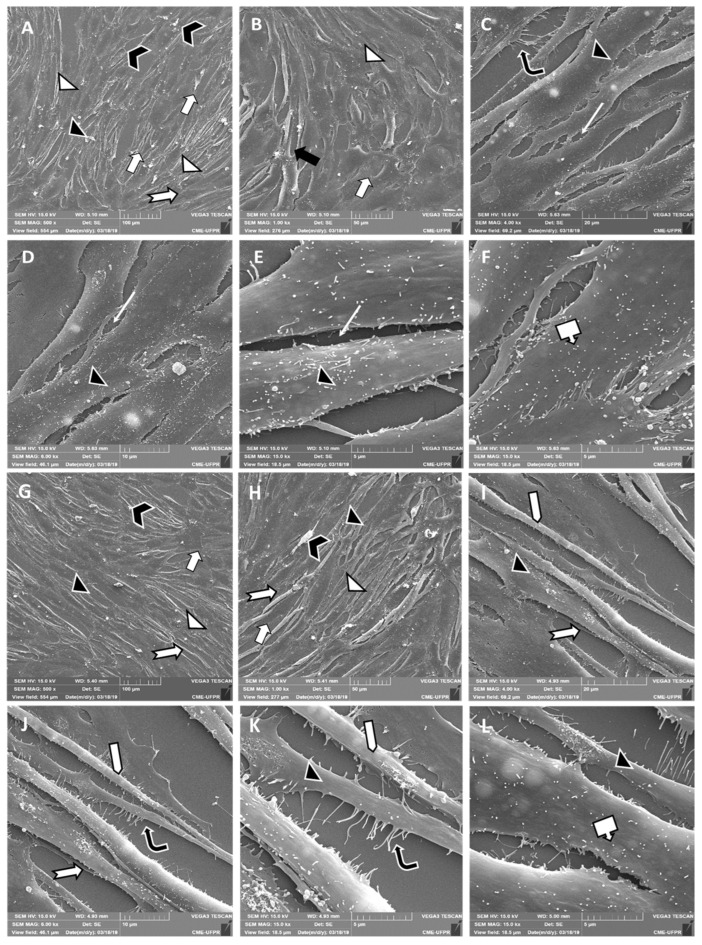

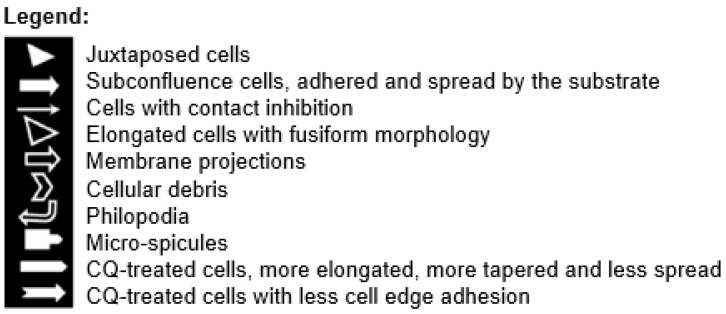
Ultrastructural differences between control cells and CQ-treated cells via SEM. Images (**A**–**F**) represent control cells maintained in cell culture for 17 days only in the presence of fetal bovine serum and medium (14-day maintenance period to induce cell differentiation). The (**G**–**L**) images show cells maintained in culture for 14 days only in the presence of medium and fetal bovine serum for cell differentiation, later exposed to CQ (1 µg/mL) for a 72 h period. Images (**A**,**G**) are enlarged at 500×, (**B**,**H**) at 1000×, (**C**,**I**) at 4000×, (**D**,**J**) at 6000×, and (**E**,**F**,**K**,**L**) at 15,000×.

In order to observe morphological and ultrastructural changes in podocytes induced via lysosomal accumulation, SEM was performed (Figure 4).

Figure 4A–F depict control cells (cultured media alone). In Figure 4A,B, these cells exhibit an organized arrangement without overlap, indicating inhibited contact. They adhere well to the substrate, presenting an elongated, spindle-shaped morphology with consistent membrane projections. Figure 4A,B also show some cell debris, likely a result of prolonged culture, suggesting potential cell death or membrane debris. Figure 4C provides a closer view of characteristic membrane projections known as filopodia. In Figure 4C–E, microspheres are evident on the cell surface, with close contact and adhesion to the substrate.

Figure 4G–L show the cells exposed to CQ. In Figure 4G,H, the cells have morphological patterns and culture organization similar to those observed for the control cells. Notably, CQ-treated cells are adherent and spread out, with an elongated morphology and a fusiform pattern. Additionally, these cells are not stacked on top of each other, demonstrating contact inhibition. Cell debris is also observed throughout the cell culture. In Figure 4I,J, it is evident that the cell bodies are losing adhesion to the substrate, with cells appearing less spread out and less adhered. Figure 4J,K also show cells with less lateral expansion of the cell body and an elongated, tapered, and narrow morphology.

In summary, the treatment with CQ via ultrastructural analysis shows cells with a loss of adhesion and a lower degree of spreading and lateral expansion of the cell body when compared to control cells.

**Figure 5:**
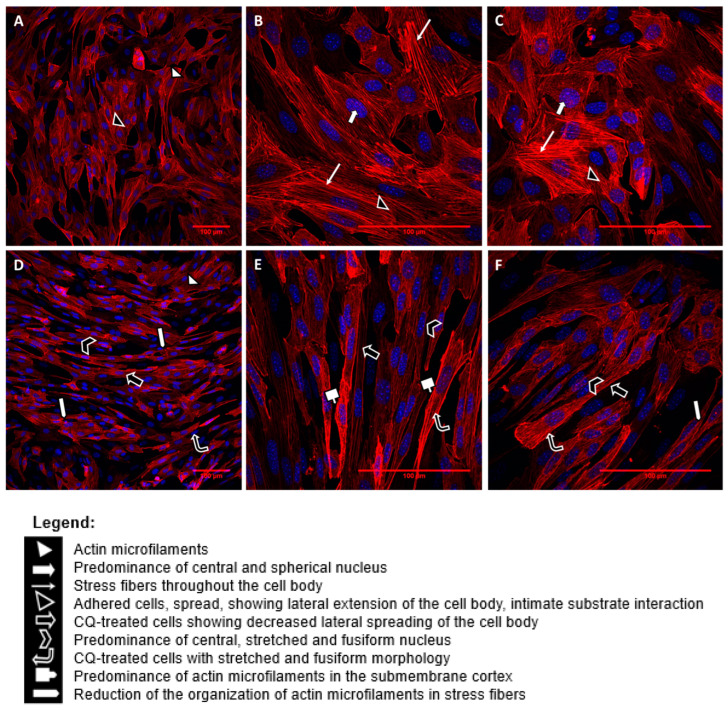
Fluorescence analysis of CQ-treated cells under confocal microscopy. Images (**A**–**C**) represent control cells maintained in cell culture for 17 days only in the presence of fetal bovine serum and medium (14-day maintenance period to induce cell differentiation). Images (**D**–**F**) show cells maintained in culture for 14 days only in the presence of medium and fetal bovine serum for cell differentiation. Subsequently, they were exposed to QC (1 µg/mL) for a period of 72 h. Images (**A**,**D**) are 200× magnified, and images (**B**,**C**,**E**,**F**) are 600× magnified.

In order to evaluate the F-actin cytoskeleton after treatment of cells with CQ, the staining was performed with the ActinRed™ 555 Rhodamine phalloidin probe, as can be seen in Figure 5.

Figure 5A–C display images of control cells, while Figure 5D–F depict cells treated with CQ, as described previously. In Figure 5A–C, control cells exhibit intense marking and organized actin microfilaments. The nuclei of these cells are spherical and centrally located within the cell body, showcasing a clear organization of stress fibers throughout. These cells, elongated and spindle-shaped, demonstrate adherence and substantial interaction with the substrate. They are either juxtaposed or sub-confluent. Interestingly, Figure 5D–F reveal a noticeable change in the lateral spreading of cell bodies after exposure to CQ. The nuclei remain centrally located but undergo morphological changes, becoming visibly elongated and following the cell’s overall shape. The cells appear significantly more elongated and spindle-shaped, displaying intense markings of predominantly deposited actin microfilaments. Notably, the submembrane cortex shows a less intense organization of microfilaments in stress fibers throughout the cell body. Actin microfilaments are now present in reduced form in long filaments due to the extension of the cell body. In this context, the cells lose their ability to organize stress fibers for lateral expansion, resulting in reduced spreading and lateral adhesion to the substrate.

The CQ treatment in the podocytes evidently induced loss of adhesion, a lower degree of spreading, and decreased organization of stress fibers, inducing more elongated, fusiform cells with less expansion of the cell body.

### 2.4. Effects of CQ on α3β1 Integrin

**Figure 6:**
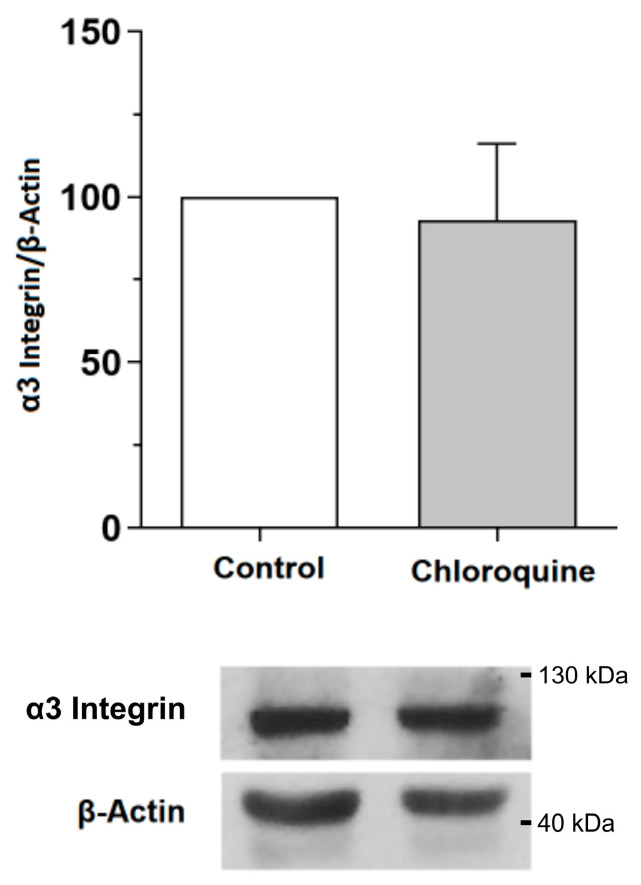
Quantitative analysis of α3 integrin via Western Blotting. Analysis of α3 integrin protein extracts in untreated (control) or CQ-treated (1 µg/mL) podocytes for 72 h at 37 °C. The values refer to the mean ± SEM of three independent experiments (*n* = 3). The data were analyzed using the Mann–Whitney test (*ns* > 0.05).

The gene expression of the α3 and β1 integrin chains was evaluated using RT-qPCR in podocytes treated or not with CQ (1 µg/mL) at 37 °C for 72 h. The evaluated genes were *Itgb1* and *Itga3*, representing each of the α3β1 integrin chains, respectively. Our data showed no statistical difference between treatment groups for both integrin genes (Figure S1). Also, the protein expression of the α3 integrin chains was evaluated via Western Blotting, in which no statistical difference was observed between podocytes treated or not with CQ (Figure 6).

**Figure 7:**
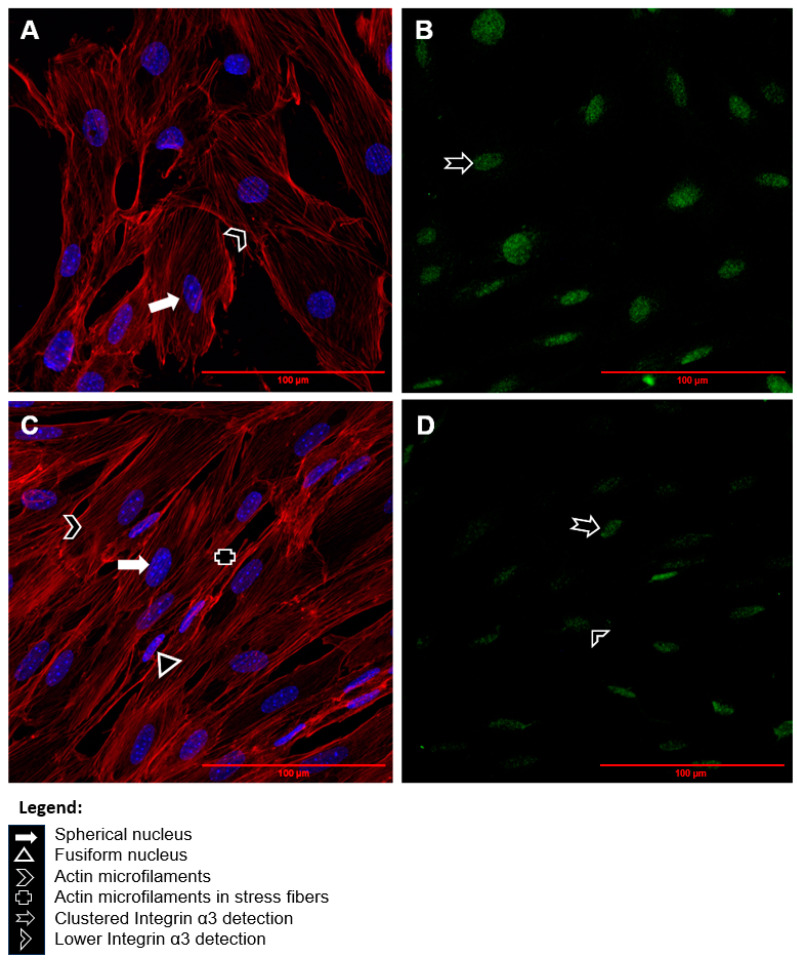
Fluorescence analysis of α3 integrin under confocal microscopy. Images (**A**,**B**) represent control cells. Images (**C**,**D**) represent cells exposed to QC (1 µg/mL) for a period of 72 h. The (**B**,**D**) images show clustered α3 integrin with lower detection after exposition to CQ. The nucleus (blue) was marked with DAPI, the F-actin cytoskeleton (red) was marked with the ActinRed™ 555 Rhodamine phalloidin probe, and α3 integrin (green) was marked with the Alexa Fluor^®^ 647 Phalloidin probe, respectively. All images are 600× magnified.

In order to evaluate the cellular distribution of α3 integrin after treatment of cells with CQ, an immunofluorescence assay was performed. Figure 7A,B depict control cells, while Figure 7C,D display images of cells treated with CQ, as described previously. In Figure 7A, spherical nuclei and actin microfilaments are observed. However, elongated cells with fusiform nuclei and actin microfilaments in stress fibers are observed after exposure to CQ (Figure 7C). Notably, Figure 7B–D reveal the detection of clustered α3 integrin, with reduced labeling in cells that received treatment with CQ (Figure 7D).

## 3. Discussion

Podocytes are highly specialized epithelial cells that cover the glomerular basement membrane (GBM) through their cell extensions called pedicels [19]. These cells are a very important component in the glomerular filtration barrier (GFB), as they are involved in the synthesis of GBM and interact with the endothelium to maintain its viability, thus forming a structure in which the blood filtration process occurs [20].

The present study shows that the CQ can reduce the cell’s viability in a dose-dependent manner (Figure 1) and, at a sub-lethal concentration (1.0 µg/mL), promotes lysosomal accumulation (Figure 2 and Figure 3). This result corroborates what has already been described for patients who make chronic use of this medication and develop signs and symptoms of kidney impairment, as occurs in FD [11]. As a result of this cellular dysfunction, podocytes treated with CQ showed changes in cell morphology, as shown in Figure 4 and Figure 5. Podocyte morphology, including its cell extensions, is all sustained based on the actin-F cytoskeleton, and its structure is essential for these cells to play their role in GFB.

Utilizing immortalized endothelial cells, Inagaki et al. demonstrated that CQ’s specific reduction in α-GAL activity resulted in ultrastructural changes such as the formation of lysosomal inclusions of glycosphingolipids [21]. More recently, we demonstrated the same phenomenon in endothelial cells, albeit at half the CQ concentration used in the prior study [22]. CQ is acknowledged for altering lysosomal properties, including increased pH and changes in membrane permeability and enzyme activity [11,16,17]. Cellular lipid accumulation is recognized to initiate a cascade of proinflammatory and profibrotic pathways in FD, leading to cellular structural changes, tissue damage, and ultimately, organ dysfunction [23]. Renal impairment stands out as a primary contributor to death and disability in FD, and podocyte dysfunction is one of the main causes of Fabry nephropathy [6,19,24].

Due to cellular dysfunction, podocytes treated with CQ exhibited alterations in both morphology and structure. Our findings align with Kang et al.’s (2020) study, reporting disruption of the actin cytoskeleton and reduced motility in human podocytes treated with 25 µM of CQ for 24 and 48 h [25]. This study also identified differential expression of lysosome-related proteins and cell adhesion molecules in CQ-exposed podocytes, potentially influencing cell stability [25]. The maintenance of podocyte morphology, including cell extensions, relies on the F-actin cytoskeleton, a structure crucial for the proper functioning of the glomerular filtration barrier (GFB). Previous studies have demonstrated that mutations in genes encoding proteins responsible for linking the F-actin cytoskeleton with focal adhesion proteins can result in podocyte damage, the thinning of pedicels, proteinuria, and the development of a glomerular disorder [19,26,27]. Given the significance of podocyte morphology, alterations in the F-actin cytoskeleton may have serious implications for the functions of these cells.

The α3β1 integrin is the major protein that links the actin-F cytoskeleton with the GBM and, consequently, is the most important protein that regulates the podocyte’s focal adhesion [18,28]. We focused on assessing the expression of α3 integrin, which is known to have a significant effect on podocyte adhesion [18,28]. Our data did not indicate significant changes in gene expression and protein levels in podocytes in the presence of CQ with RT-qPCR and Western Blotting; although there was less detection of α3 integrin in immunofluorescence, as can be seen in Figure 7. The protein reduction in α3 integrin demonstrated in the exposed results reinforces its direct relationship with the structural abnormality of podocytes, a fact that may contribute to nephropathy in patients with FD. These results demonstrate that CQ presents toxicity for these cells and, consequently, can impair kidney function. However, protein levels and the cellular distribution of β1 integrin remain yet to be analyzed. The dysregulation in the gene expression of these two chains also occurs in patients with diabetic nephropathy (DN), who present the following symptoms: podocyte detachment of the GBM, erasure of the pedicels in renal biopsy, podocyturia, microalbuminuria, and proteinuria [29,30,31,32]. In vivo studies showed that disorders targeting α3 integrin, such as mutation and deletion, can, respectively, prevent the formation of pedicels in podocytes, compromising glomerular development and causing glomerular injury in mice [33,34]. Also, Chen et al. have demonstrated that there is an in vitro decrease in the protein expression of α3β1 integrin as well as a decrease in the capacity of podocyte adhesion in DN [35]. Therefore, the α3β1 integrin deregulation may be related to glomerulopathies such as the congenital nephrotic syndrome [36,37].

We recognize that the present study has some limitations. The podocyte model used for the tests was murine because of the difficulty in obtaining human podocytes. In addition, we evaluated the α3β1 gene expression and only the protein expression of the α3 subunit. Evaluation of β1 subunit expression would be the next step to investigate the involvement of deregulation of this heterodimer in this pathway. This is expected to provide valuable insights into the mechanism that leads to the GBM detachment of podocytes in FD.

## 4. Conclusions

The main findings of the present study reveal that treatment with CQ at sub-lethal doses (1.0 µg/mL) can induce alterations in podocytes, including lysosomal lipid accumulation (mimicking FD), cytoskeletal remodeling, and morphological and structural changes. In essence, we propose a podocyte model for the study of FD. Moreover, the expression profile of the primary focal podocyte adhesion integrin in response to lysosomal accumulation was demonstrated. The same profile is observed in DN and in vivo data, also indicating a parallel progression of these two diseases. Consequently, the results obtained present potential avenues for new therapeutic targets aimed at enhancing the quality of life for these patients.

## 5. Materials and Methods

### 5.1. Reagents

RPMI 1640 medium (RPMI), fetal bovine serum (FBS), collagen I from rat tail, and Penicillin/Streptomycin were purchased from Gibco (Grand Island, NE, USA). Fluoromount G and DAPI were obtained from Life Technologies (Carlsbad, CA, USA). LysoTracker^®^ DND-99 probe, Alexa Fluor^®^ 647 Phalloidin, and Actin Red™555 ReadyProbes™ Rhodamine phalloidin were commercially obtained (Thermo Scientific, Waltham, MA, USA). Murine Interferon Gamma (IFN-γ) was purchased from Prepotech. [4, 5-dimethyl-thiazol-2-yl]-2, 5-diphenyltetrazolium bromide (MTT), neutral red solution (NR), and dimethyl sulfoxide (DMSO) were obtained from Sigma-Aldrich (St. Louis, MO, USA). CQ was purchased from Cristália (São Paulo, Brazil). Western Blotting reagents were obtained from GE HealthCare Life Sciences (Little Chalfont, UK). All other reagents were obtained from Sigma-Aldrich (St. Louis, MO, USA) if not otherwise specified.

### 5.2. Podocyte Cell Culture and Treatment Conditions

The immortalized murine kidney podocyte cell line E11 obtained from Cell Lines Service (Eppelheim, Germany) was kindly provided by Prof. Dr. Niels Olsen Saraiva Câmara (Federal University of São Paulo—UNIFESP). For the podocyte culture, a collagen I matrix (0.5 mg/mL) should be made on the plates. The undifferentiated cells were grown under permissive conditions in RPMI 1640 medium supplemented with 10% FBS and IFN-γ at a concentration of 20–50 IU mL, maintained at 33 °C in a humidified atmosphere containing 5% CO_2_. After reaching about 70–80% confluence, the cells were exposed to a non-permissive condition to start the cell differentiation process. In this phase, the cells were placed in an atmosphere at 37 °C and 5% CO_2_, with a culture medium without INF-γ. After 14 days of cultivation, cells were ready for experiments [38,39]. In the sequence, the cells were exposed to CQ [21].

### 5.3. Cell Viability Assay

Cell viability was assessed using the MTT assay [40]. Podocytes were plated into 96-well culture plates at a density of 1 × 10^3^ cells per well. After 24 h of incubation, the medium was replaced, and the cells were treated with CQ for 72 h. Then, this medium was replaced with fresh medium (100 μL/well), and 10 μL of MTT (Sigma-Aldrich, Hercules, CA, USA) solution (5 mg/mL in D-PBS) was added to each well. The plate was incubated for 4 h at 37 °C. Subsequently, the media was removed and replaced with DMSO to dissolve the crystals of reduced formazan, and the absorbance was then measured at 570 nm in a Bio-Rad 680 microplate reader (CA, USA). Four experiments were performed in duplicate.

### 5.4. Acid Organelle Accumulation Assay

Podocytes were plated into 96-well plates (1 × 10^3^ cells/well) and treated with CQ (1 μg/mL) or vehicle control (culture medium) for 72 h. Next, all medium was removed, and 100 μL of NR solution (40 μg/mL) was added. Subsequently, the plate was incubated for 3 h at 37 °C and 5% CO_2_. Finally, the NR-containing medium was removed, the cells were washed with 150 μL of PBS per well, and 150 μL of distain solution (1% glacial acetic acid and 48% ethanol) was added. The absorbance was measured using a 540 nm filter spectrophotometer [41]. NR stains lysosomes as color is pH-dependent. After the NR assay, the solution was removed from the wells and washed once with PBS (200 μL/well). Next, 100 μL of violet crystal (VC) solution (0.25 mg/mL) was added per well, and the plates were incubated for 20 min at room temperature (22 °C). Then, the wells were washed 2× with PBS and solubilized with 33% acetic acid. Finally, the absorbance was measured using a spectrophotometer at a wavelength of 570 nm. The result was obtained by normalizing the NR test to the VC. Three experiments were performed in duplicate.

### 5.5. Fluorescence Lysosome Inclusions Visualization Assay

The LysoTracker^®^ DND-99 fluorescent probe was used for visualization of acid organelle inclusions such as lysosomes since lysosome size is related to the number of inclusions. The cells were plated on coverslips and treated with CQ at 1.0 µg/mL. The treatment medium was removed, and cells were washed twice with sterile PBS. Next, cells were incubated for 90 min at 37 °C with 50 nM of LysoTracker^®^ DND-99 probe diluted in culture medium and subsequently washed twice with PBS, fixed with 2% paraformaldehyde for 20 min, and washed again with PBS. The coverslips were mounted on histological slides with Fluoromont-G^TM^ with DAPI mounting medium, sealed with formaldehyde-free color enamel, and observed under a Nikon A1RSiMP confocal microscope (NIKON, Tokyo, Japan). Three experiments were performed in duplicate.

### 5.6. Actin-F Cytoskeleton and α3 Integrin Visualization Assay

The Alexa Fluor^®^ 647 Phalloidin fluorescent probe was used for visualization of integrin α3 localization, and the ActinRed™ 555 Rhodamine phalloidin probe was used for visualization of the actin-F cytoskeleton. The cells were plated on coverslips and treated with CQ at a 1.0 µg/mL concentration, as described before. Subsequently, the medium was removed, and the coverslips were washed twice with PBS. The cells were fixed with 2% paraformaldehyde (PFA) diluted in PBS for 20 min at room temperature (22 °C). After fixation, the cells were washed twice with PBS and incubated with integrin α3 antibody (1:1000, Thermo Fisher Scientific, Waltham, MA, USA) overnight at 4 °C. Alexa Fluor^®^ 647 Phalloidin was diluted in a solution of 0.01% saponin in PBS and added for 1 h. Posteriorly, the cells were incubated with ActinRed™ 555 Rhodamine phalloidin for 45 min. The coverslips were mounted on a histological slide containing Fluoromont g with DAPI. After this process, the coverslips were sealed with formaldehyde-free color enamel, and visualization was performed using a Nikon A1RSiMP confocal microscope (NIKON, Tokyo, Japan). Three experiments were performed in duplicate.

### 5.7. Integrins α3 and β1 Gene Expression

The total RNA of podocytes was isolated from cells lysed with Trizol (Invitrogen, Carlsbad, CA, USA), according to the manufacturer’s recommendations. The purity and concentration of the RNA were verified with the 260/280 nm ratio measured on the NanoDrop 2000 spectrophotometer (Thermo Scientific, Waltham, MA, USA). The RNA integrity was analyzed via agarose gel electrophoresis. The mRNA molecules were converted into complementary DNA (cDNA) using the High-Capacity RNA-to-cDNA kit (Applied Biosystems, Waltham, MA, USA). For RT-qPCR, the cDNA was amplified with specific primer oligonucleotides using the SYBRTM Green PCR Master Mix (Thermo Scientific, Waltham, MA, USA) in the StepOne PlusTM Real Time PCR System (Thermo Scientific, Waltham, MA, USA) thermocycler. The relative expressions of the genes were analyzed using the 2^−ΔΔCT^ method. The genes analyzed were *Rplp0* (ribosomal protein lateral stalk subunit P0), *Itgb1* (Integrin Subunit Beta 1), and *Itga3* (Integrin Subunit Alpha 3). The sequences of the oligonucleotides initiating the target genes are described in Table 1. Five experiments were performed in duplicate.

### 5.8. Analysis of Integrin α3 Levels via Western Blotting

The cells were cultured (3 × 10^5^ cells/dish) in 100 mm dishes and were stimulated for 72 h with CQ at a concentration of 1.0 µg/mL for 72 h. The cells were washed with ice-cold PBS and lysed in 100 μL of radioimmunoprecipitation assay buffer (50 mM sodium chloride, 1.0% Triton X-100, 0.5% sodium deoxycholate, 0.1% SDS, 50 mM Tris, pH 8.0) for 20 min. The cell lysates were centrifuged for 20 min at 12,000× *g* at 4 °C, and the supernatants were collected. Equal amounts of protein (30 μg) were separated using 8% sodium dodecyl sulfate-polyacrylamide gel electrophoresis and transferred to nitrocellulose membranes (GE HealthCare Life Sciences, Little Chalfont, UK). The membranes were blocked for 1 h at room temperature with Tris-buffered saline containing 0.05% Tween 20 (TBS-T) and 3% casein. After washing with TBS-T, the membranes were incubated overnight with the integrin α3 antibody (1:1000, Thermo Fisher Scientific, Waltham, MA, USA) at 4 °C with gentle shaking. The primary antibodies were detected using horseradish peroxidase-conjugated goat anti-rabbit IgG and visualized using enhanced chemiluminescence Western Blotting reagents (GE HealthCare Life Sciences, Little Chalfont, UK). Band intensity was analyzed using Software Image Studio Lite 5.2 (Lincoln, NE, USA). All analyses were performed in triplicate.

### 5.9. Morphological and Ultrastructural Analysis Performed via a Scan Electronic Microscopy

The SEM was the technique used for the evaluation of possible morphological changes and was ultra-adapted through the accumulation of lysosomes in the podocytes. The cells were plated (1 × 10^5^ cells/coverslips) in circular coverslips (13 mm in diameter) at a 24-well plate and were treated with CQ at a concentration of 1.0 µg/mL for 72 h. After treatment, the medium was removed, and the cells were washed twice with PBS and fixed with Karnovski’s solution (2% glutaraldehyde, 4% paraformaldehyde, 0.1 M of CaCl_2_, and pH 7.4) for 1 h. Subsequently, they were washed with 0.1 M of sodium cacodylate buffer (pH 7.4) and post-fixed with 1% osmium tetroxide (diluted in 0.1 M of sodium cacodylate buffer, pH 7.4) for 1 h. After the fixation, the cells were washed in 0.1 M of sodium cacodylate buffer and dehydrated by increasing ethanol engines (30%, 50%, 70%, 90%, and twice with 100%) for a period of 10 min at each concentration. Next, the cells were subjected to the critical point in the CPD 010 device (Critical Point Dryer, Bal-Tec AG, Balzers, Liechtenstein) 030-Balzers, metalized with gold in the device (SCD 030-Balzers, Balzers Union, Balzers, Liechtenstein), and analyzed using a scanning electron microscope (Tescan-Vega3-LMU-Scanning electron microscope, Kohoutovice, Czech Republic) at the Center for Electronic Microscopy of Federal University of Paraná (UFPR). The images were captured in increments of 500×, 1000×, 4000×, 6000×, and 15,000×. Four experiments were performed in duplicate.

### 5.10. Data Analyses

Statistical analyses were performed using the statistical package JMP (version 8.0; SAS Institute Inc., Cary, NC, USA). Comparisons between groups were performed using a Student’s *t*-test or an analysis of variance (ANOVA) with Dunnett’s multiple comparison test for paired data and Mann–Whitney for unpaired data. Values were expressed as the mean ± standard error of the mean (SEM). Three to five independent experiments were performed. *p* < 0.05 was considered statistically significant.

## Figures and Tables

**Table 1:** Primer oligonucleotides used in RT-qPCR.

Gene	Oligonucleotides *
*Itgb1*	5′-AACTTGTTGGTCAGCAACGC-3′ (F) 5′-AACCGCAACCTGCATGATTG-3′ (R)
*Itga3*	5′-CCTCTTCGGCTACTCGGTC-3′ (F) 5′-CCGGTTGGTATAGTCATCACCC-3′ (R)
*Rplp0*	5′-CGACCTGGAAGTCCAACTAC-3′ (F) 5′-ACTTGCTGCATCTGCTTG-3′ (R)

* F, forward; R, *reverse.*

## Data Availability

The data presented in this study are available on request from the corresponding author.

## Supplementary Materials

The following supporting information can be downloaded at https://www.mdpi.com/article/10.3390/toxins15120700/s1, Figure S1: Relative expression of the *Itga3* and *Itgb1* via RT-qPCR. Increased relative expression of the *Itga3* and *Itgb1* genes in untreated (control) or CQ-treated (1 μg/mL) podocytes for 72 h at 37 °C. The relative expression of mRNA was determined with the 2^−ΔΔCt^ method, using *Rplp0* as a normalizing gene. The values refer to the mean ± SEM of three independent experiments (*n* = 3). The data were analyzed using the Mann–Whitney test (*ns* > 0.05).
