# Supplementary material for: The Role of α3β1 Integrin Modulation on Fabry Disease Podocyte Injury and Kidney Impairment
Source: Toxins (Basel). 2023 Dec 14;15(12):700. doi: 10.3390/toxins15120700 (PMC10748128; doi:10.3390/toxins15120700)
Supplement: Supplementary file 1 [file toxins-15-00700-s001.zip › toxins-2674880-supplementary.pdf]

Supplementary material

Supplementary Figure S1

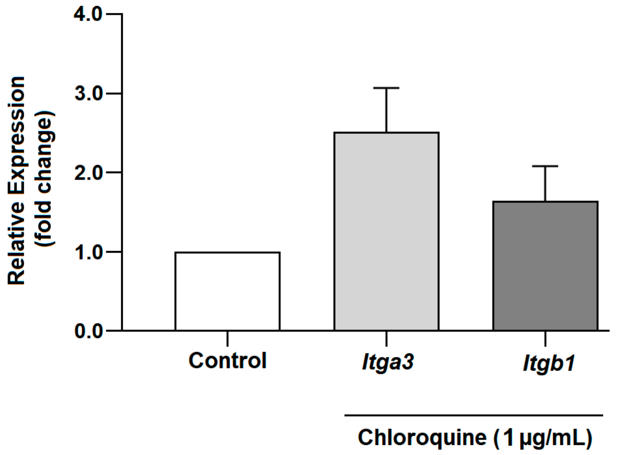

Figure S1. Relative expression of the *Itga3* and *Itgb1* by RT-qPCR. Increased relative expression of the *Itga3* and *Itgb1* genes in untreated (control) or CQ-treated (1 µg/mL) podocytes for 72 h at 37°C. The relative expression of mRNA was determined by the 2 − ΔΔCt method, using *Rplp0* as a normalizing gene. The values refer to the mean ± SEM of three independent experiments (n = 3). The data were analyzed using the Mann-Whitney test (*ns*>0.05).
